# Clinical Features in Children With Kawasaki Disease Shock Syndrome: A Systematic Review and Meta-Analysis

**DOI:** 10.3389/fcvm.2021.736352

**Published:** 2021-09-21

**Authors:** Zhimin Zheng, Yanzhi Huang, Zhiyi Wang, Jia Tang, Xiaoqian Chen, Ying Li, Meng Li, Chengye Zang, Yibo Wang, Liwu Wang, Yingwei Ma, Liwei Sun

**Affiliations:** ^1^College of Clinical Medicine, Changchun University of Chinese Medicine, Changchun, China; ^2^Jilin Children's Medical Center, Children's Hospital of Changchun, Changchun, China

**Keywords:** Kawasaki disease, shock, Kawasaki disease shock syndrome, clinical feature, meta-analysis

## Abstract

**Objective:** This study aimed to identify the clinical features of Kawasaki disease shock syndrome (KDSS) in children.

**Methods:** The case-control studies of KDSS and KD children up until April 30, 2021 were searched in multiple databases. The qualified research were retrieved by manually reviewing the references. Review Manager 5.3 software was used for statistical analysis.

**Results:** The results showed that there was no significant difference in the incidence of male and female in children with KDSS. Children with KDSS compared with non-shocked KD, there were significant difference in age, duration of fever, white blood cell (WBC) count, percentage of neutrophils (NEUT%), platelet count (PLT), c-reactive protein level (CRP), alanine transaminase concentration (ALT), aspartate transaminase concentration (AST), albumin concentration (ALB), sodium concentration (Na), ejection fraction, and length of hospitalization as well as the incidence of coronary artery dilation, coronary artery aneurysm, left ventricular dysfunction, mitral regurgitation, pericardial effusion, initial diagnosis of KD, intravenous immunoglobulin (IVIG) resistance and receiving second dose of IVIG, vasoactive drugs, hormones, and albumin. In contrast, there was no difference in the hemoglobin concentration, erythrocyte sedimentation rate, and the incidence of conjunctival injection, oropharyngeal change, polymorphous rash, extremity change, and incomplete KD.

**Conclusion:** Current evidence suggested that the children with KDSS had more severe indicators of inflammation and more cardiac abnormalities. These patients were resistant to immunoglobulin treatment and required extra anti-inflammatory treatment.

**Systematic Review Registration:** PROSPERO registration number CRD42021241207.

## Introduction

Kawasaki disease (KD) is an acute immune systemic small- to medium-sized vasculitis characterized by fever, cervical lymphadenopathy, conjunctival injection, oropharyngeal changes, polymorphous rash, and extremity changes. Coronary artery lesion (CAL) is the most serious complication (1). In recent years, children with KD have developed serious complications, such as shock and heart failure, and Kanegaye et al. (2) defined this hemodynamically unstable KD as Kawasaki disease shock syndrome (KDSS), with the specific criteria of the presence of a systolic blood pressure consistently below 20% of the mean systolic blood pressure in normal children of the same age, or signs of impaired perfusion in the peripheral circulation. Most of the studies in KDSS in China and abroad are case reports or the retrospective clinical analyses of small samples, and the descriptions of its clinical features vary from one another. A study with the largest sample size in China came from Taiwan, with an incidence of KDSS in KD being 1.45% (3), which was much lower than the reported ~2.8–5.3% in the United States (4). Thus, there is a lack of a large sample and multicenter study of KDSS in mainland China, and the understanding of the clinical KDSS features is not sufficient. In this study, we had conducted an evidence-based analysis of controlled studies on the clinical characteristics of KDSS in children in the past 10 years, aiming at deepening the understanding of KDSS, summarizing the experience, and improving the clinical diagnosis and treatment activities.

## Methods

### Search Strategy

By utilizing medical subject headings (MeSH) or Emtree combining with keywords, we searched in databases, such as PubMed, Embase, Web of Science, CNKI (China National Knowledge Infrastructure), VIP (Chinese Scientific Journals Full-Text Database), CBM (China Biological Medicine Database), and Wanfang database. We searched on the websites from the database built until April 30, 2021. The search terms used were ‘Kawasaki disease,' ‘mucocutaneous lymph node syndrome,' ‘Kawasaki disease shock syndrome,' and ‘shock.' The search strategy on PubMed was “Mucocutaneous Lymph Node Syndrome”[Mesh] and “Shock”[Mesh].

### Inclusion Criteria

(1) Case-control study of KDSS vs. KD without shock in children. (2) The diagnosis of KD and incomplete KD (IKD) according to American Heart Association common standards (1, 5). (3) The diagnostic criteria of KDSS also referred to Kanegaye et al. (2).

### Exclusion Criteria

(1) Duplicated data; (2) reviews, conference abstracts, and case reports; (3) mean value and SD were not directly or indirectly provided; and (4) popular science journals.

### Data Extraction

Two researchers independently selected eligible studies based on the predetermined inclusion and exclusion criteria. The following data were extracted: (1) General information: first author, year of publication, sample size, gender, and age. (2) Kawasaki features: duration of fever, cervical lymphadenopathy, conjunctival injection, oropharyngeal changes, polymorphous rash, and extremity changes; (3) Laboratory examinations: white blood cell count (WBC), percentage of neutrophils (NEUT%), hemoglobin concentration (HGB), platelet count (PLT), c-reactive protein level (CRP), erythrocyte sedimentation rate (ESR), alanine transaminase concentration (ALT), aspartate transaminase concentration (AST), albumin concentration (ALB), and sodium concentration (Na); (4) Ultrasound cardiogram (UCG): Coronary artery dilation, coronary artery aneurysm, ejection fraction, left ventricular dysfunction, mitral regurgitation, and pericardial effusion. (5) Diagnosis and treatment: initial diagnosis of KD, IKD, the second dose of Intravenous Immunoglobulin (IVIG), IVIG resistance, receiving vasoactive drugs, receiving hormones, receiving albumin, and length of hospitalization. The mean ± SD was calculated when the median and interquartile range (IQR) were provided (6–9). If the same study data appeared in different articles, only one article with complete data was selected. The disagreements were resolved through the discussion or by third-party adjudication.

### Quality Assessment

The quality of each included study was surveyed utilizing the Newcastle-Ottawa Scale (NOS). The total score was nine (Literature with the total score ≥7 is defined as high-quality, 3–7 as medium-quality, and <3 as low-quality). Any disagreements were resolved through discussion or by third-party adjudication.

### Statistical Analysis

Data analysis was performed with Review Manager 5.3. The statistical heterogeneity was evaluated by *I*^2^, and the effect model was selected according to the results. Dichotomous data were analyzed with OR (Odds Ratio) and 95% *CI*. Continuous variables were analyzed with WMD (weighted mean difference) or SMD (standard mean difference) and 95% CI. *P* < 0.05 indicated a statistical significance. Egger's test was used to test for publication bias. Publication bias was determined when Egger's test showed a *P*-value < 0.1.

## Results

### Overview of the Included Studies

**Figure 1:**
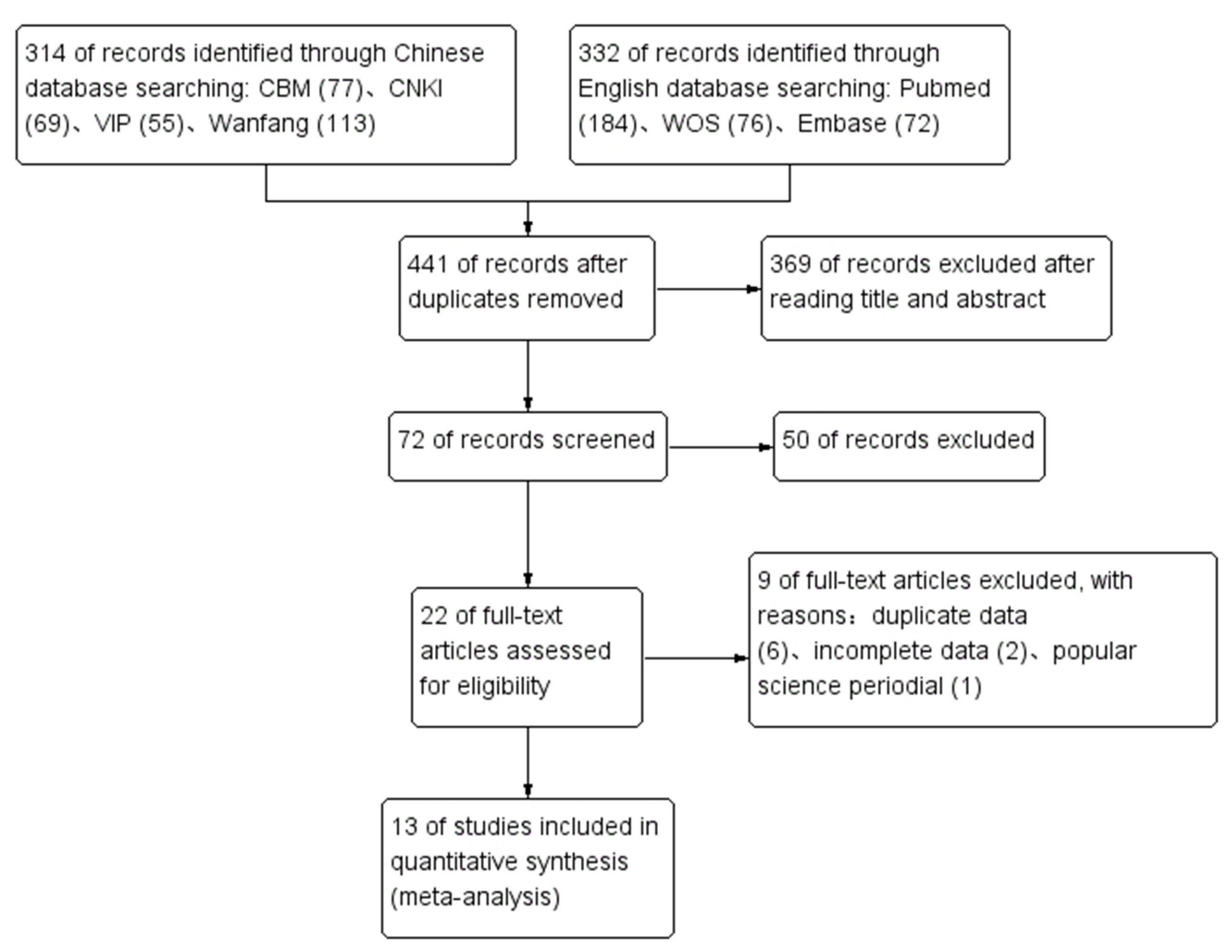
Flow diagram for the process of searching and selecting.

Among the identified 646 studies, and 13 studies were ultimately included (2, 10–21), containing 200 children with KDSS and 958 children with KD. Among 200 children with KDSS, 106 were boys (53%) and 94 were girls (47%). In the 13 studies, the two studies were considered as moderate quality, while the other 11 studies were qualified as high quality. The process of searching and selecting is outlined in Figure 1. The characteristics of the included studies are summarized in Table 1.

**Table 1:** Characteristics of the included studies.

**References**	***N* (KDSS/KD)**	**Male (KDSS/KD)**	**Age**		**Kawasaki features**	**Laboratory examinations**	**UCG**	**Diagnosis and treatment**	**NOS**
			**KDSS**	**KD**					
Kanegaye (2)	13/174	4/104	2.8 (2.2~5.9) (y)	2.1 (0.9~3.9) (y)	Ψ	1, 2, 4, 6	1, 2, 3, 4, 5	Ψ	7
Gámez-González (10)	11/203	8/126	42 (3~120) (m)	23 (2~186) (m)	Ψ	Ψ	6	1, 4	7
Chen (11)	9/27	3/16	3.2 ± 3.3 (y)	2.0 ± 1.8 (y)	1	1, 2, 4, 5, 6, 7, 8, 9	1	1, 3, 5, 7, 8	8
Taddio (12)	5/79	4/49	25 (5~139) (m)	27 (18~44) (m)	Ψ	3, 6	1, 2, 4, 5, 6	2, 3, 5, 6, 8	7
Schuster (13)	12/36	4/26	58.5 ± 27.3 (m)	40.1 ± 34 (m)	2, 3, 4, 5, 6	1, 3, 4, 5, 6, 7, 8, 9, 10	4, 5, 6	1, 4, 8	8
Qi (14)	16/30	9/22	3.95 ± 2.56 (y)	3.13 ± 1.79 (y)	1	1, 2, 4, 5, 6, 7, 9, 10	2, 3	4, 8	7
Ma (15)	21/24	11/13	4.9 ± 2.8 (y)	2.7 ± 1.9 (y)	1	1, 2, 3, 4, 5, 6, 7, 8, 9	1, 4	1, 3, 5, 6, 7, 8	6
Li (16)	27/43	17/19	43.41 ± 31.42 (m)	28.81 ± 21.51 (m)	1, 2, 3, 4, 5, 6	1, 2, 3, 4, 5, 6, 9, 10	2, 3	1, 2, 3, 4, 8	6
Zhao (17)	21/119	13/68	28 (15.5~72.0) (m)	24 (13.0~36.0) (m)	1, 2, 3, 4, 5, 6	1, 2, 3, 4, 5, 6	1, 4, 5, 6	1, 3, 6, 7	7
Du (18)	15/30	8/18	55.40 ± 31.20 (m)	49.80 ± 33.50 (m)	1	1, 2, 3, 4, 5, 6, 7, 8, 9, 10	1, 3	3, 5, 6, 7, 8	8
Su (19)	20/40	5/9	3.2 ± 1.1 (y)	3.4 ± 1.3 (y)	1, 2, 3, 4, 5, 6	1, 2, 3, 4, 5, 6, 9, 10	Ψ	Ψ	8
Park (20)	13/91	6/53	5.1 (0.5–10.6) (y)	2.3 (0.2–8.4) (y)	2, 3, 4, 5, 6	6, 9, 10	2, 3, 4	1, 2, 3, 5, 6	7
Li (21)	17/68	14/45	64.7 (49.6~90.0) (m)	25.3 (14.2~45.0) (m)	2, 3, 4, 5, 6	1, 2, 3, 4, 5, 9, 10	1, 2, 3, 4, 5, 6	1, 2, 4, 5	8

*KDSS, Kawasaki disease shock syndrome; KD, Kawasaki disease without shock*.

*Kawasaki features: 1 duration of fever; 2 Cervical lymphadenopathy; 3 conjunctival injection; 4 Oropharyngeal changes; 5 Polymorphous rash; 6 Extremity changes*.

*Laboratory examinations: 1 WBC; 2 NEUT%; 3 HGB; 4 PLT; 5 CRP; 6 ESR; 7 ALT; 8 AST; 9 Alb; 10 Na^+^*.

*UCG: 1 Coronary artery dilation; 2 Coronary artery aneurysm; 3 ejection fraction; 4 Left ventricular dysfunction; 5 Mitral regurgitation; 6 Pericardial effusion*.

*Diagnosis and treatment: 1 initial diagnosis of KD; 2 IKD; 3 second dose of IVIG; 4 IVIG resistance; 5 vasoactive drugs; 6 hormones; 7 albumin; 8 length of hospitalization*.

*NOS, Newcastle-Ottawa Scale*.

### Heterogeneity Analysis

The following indicators with high heterogeneity (*I*^2^ > 50% or *P* < 0.1) were used in the random effects model: Fever duration, WBC, NEUT%, HGB, PLT, CRP, ESR, ALT, ALB, mitral regurgitation, IKD, and receiving hormones. The following indicators with low heterogeneity (*I*^2^ ≤ 50% or *P* ≥ 0.1) were used in the fixed effect model: Cervical lymphadenopathy, conjunctival injection, oropharyngeal changes, polymorphous rash, extremity changes, AST, Na^+^, coronary artery dilatation, coronary artery aneurysm, ejection fraction, left ventricular dysfunction, pericardial effusion, initial diagnosis of KD, second dose of IVIG, IVIG resistance, receiving vasoactive drugs, receiving albumin, and length of hospitalization.

### Meta-Analysis

The results (Table 2) showed that, compared with the KD group, children in the KDSS group were characterized in the following aspects. As for the gender, there was no significant difference in incidence between male and female and children in the KDSS group. The children in the KDSS group were significantly older. In terms of Kawasaki features, children in the KDSS group had longer fever duration and higher incidence of cervical lymphadenopathy, and the differences were significant, while no significant differences were observed in the incidence of conjunctival injection, oropharyngeal changes, polymorphous rash, and extremity changes. As for the laboratory examination indexes, the levels of WBC, NEUT%, CRP, ALT, and AST, were significantly higher and the levels of PLT, ALB, and Na^+^ levels were significantly lower in the KDSS group, while there were no significant differences in the levels of HGB and ESR. Furthermore, UCG showed that significant differences were observed in a higher incidence of coronary artery dilation, coronary artery aneurysm, left ventricular dysfunction, mitral regurgitation, pericardial effusion, and in decreased ejection fraction for children in the KDSS group. Additionally, children in KDSS group had a lower incidence of KD initial diagnosis with a significant difference. Also, the incidences of receiving a second dose of IVIG, IVIG resistance, vasoactive drugs, hormones, albumin were higher, and prolonged length of hospitalization was observed with significant differences in the KDSS group, with no significant difference as for the incidence of IKD presented. Forest plots are shown in Supplementary Figures S1–S32.

**Table 2:** Results of meta-analysis.

**Projects**	**Effect size**	**95% CI**	***P*-value**	**Forest plot**
*General information*				
Male	1.11^a^	[0.83, 1.49]	0.48	Supplementary Figure S1
Age	0.70^c^	[0.42, 0.98]	<0.00001	Supplementary Figure S2
*Kawasaki features*				
Fever duration	1.63^b^	[0.79, 2.47]	0.0001	Supplementary Figure S3
Cervical lymphadenopathy	2.17^a^	[1.30, 3.64]	0.003	Supplementary Figure S4
Conjunctival injection	0.69^a^	[0.35, 1.37]	0.29	Supplementary Figure S5
Oropharyngeal change	1.71^a^	[0.79, 3.72]	0.18	Supplementary Figure S6
Polymorphous rash	1.17^a^	[0.66, 2.08]	0.59	Supplementary Figure S7
Extremity change	1.07^a^	[0.63, 1.83]	0.81	Supplementary Figure S8
*Laboratory examinations*				
WBC	3.23^b^	[0.73, 5.73]	0.01	Supplementary Figure S9
NEUT%	14.66^b^	[9.93, 19.39]	<0.00001	Supplementary Figure S10
HGB	−5.65^b^	[−12.75, 1.44]	0.12	Supplementary Figure S11
PLT	−82.84^b^	[−131.46, −34.22]	0.0008	Supplementary Figure S12
CRP	61.76^b^	[41.47, 82.05]	<0.00001	Supplementary Figure S13
ESR	−0.21^b^	[−10.22, 9.79]	0.97	Supplementary Figure S14
ALT	37.02^b^	[1.57, 72.47]	0.04	Supplementary Figure S15
AST	31.56^b^	[14.23, 48.89]	0.0004	Supplementary Figure S16
ALB	−6.85^b^	[−8.65, −5.06]	<0.00001	Supplementary Figure S17
Na^+^	−3.80^b^	[−4.52, −3.08]	<0.00001	Supplementary Figure S18
*UCG*				
Coronary artery dilation	4.20^a^	[2.50, 7.04]	<0.00001	Supplementary Figure S19
Coronary artery aneurysm	7.21^a^	[3.41, 15.24]	<0.00001	Supplementary Figure S20
Ejection fraction	−4.50^b^	[−6.11, −2.89]	<0.00001	Supplementary Figure S21
Left ventricular dysfunction	25.17^a^	[10.60, 59.77]	<0.00001	Supplementary Figure S22
Mitral regurgitation	6.49^a^	[2.35, 17.90]	0.0003	Supplementary Figure S23
Pericardial effusion	2.38^a^	[1.22, 4.64]	0.01	Supplementary Figure S24
*Diagnosis and treatment*				
Initial diagnosis of KD	0.08^a^	[0.03, 0.22]	<0.00001	Supplementary Figure S25
Incomplete KD	1.64^a^	[0.77, 3.50]	0.20	Supplementary Figure S26
Second dose of IVIG	8.61^a^	[4.48, 16.58]	<0.00001	Supplementary Figure S27
IVIG resistance	18.54^a^	[8.60, 39.96]	<0.00001	Supplementary Figure S28
Receiving Vasoactive drugs	509.67^a^	[138.37, 1877.34]	<0.00001	Supplementary Figure S29
Receiving Hormones	19.92^a^	[4.04, 98.31]	0.0002	Supplementary Figure S30
Receiving Albumin	23.07^a^	[9.54, 55.74]	<0.00001	Supplementary Figure S31
Length of hospitalization	5.42^b^	[4.58, 6.26]	<0.00001	Supplementary Figure S32

a *Odds Ratio (OR)*.

b *Weighted mean difference (WMD)*.

c *Standard mean difference (SMD)*.

### Publication Bias

**Figure 2:**
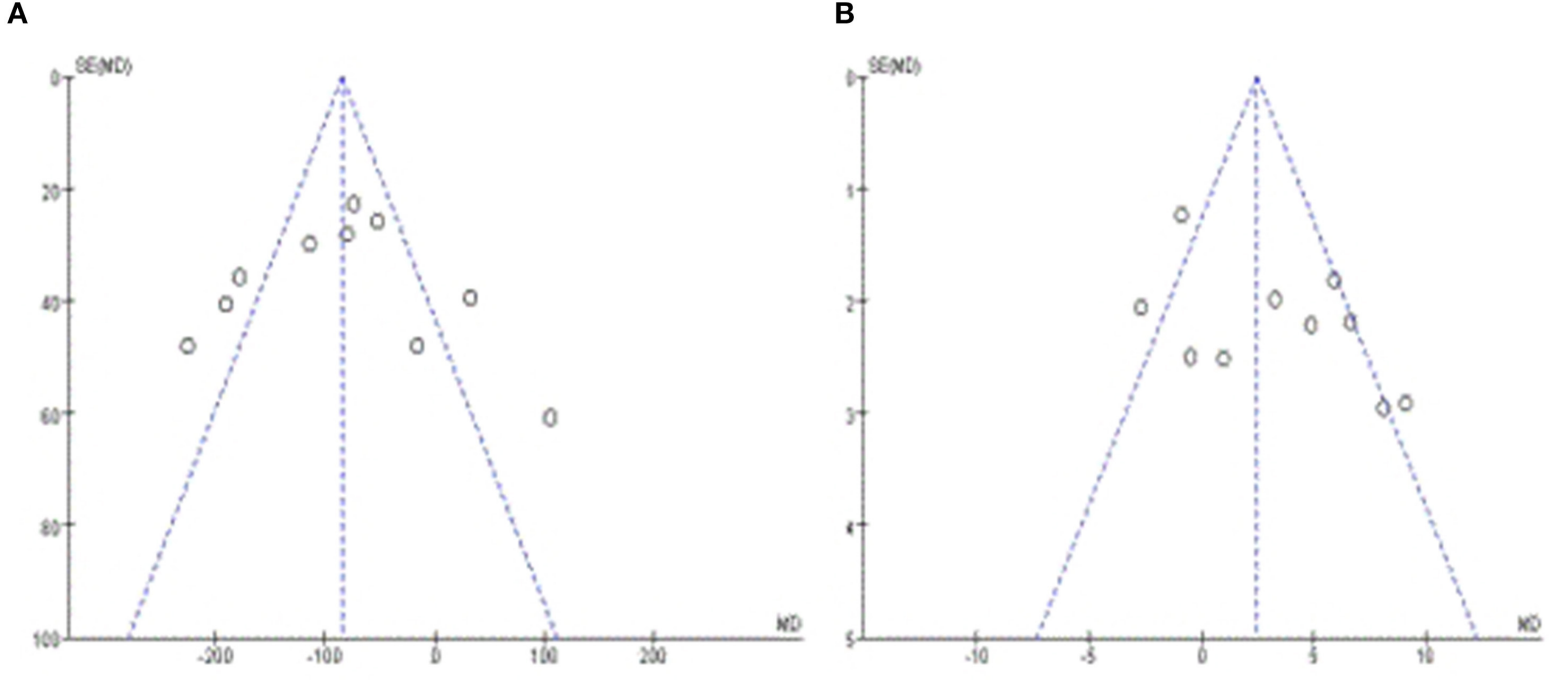
**(A)** Funnel plots of PLT. **(B)** Funnel plots of WBC.

Egger's test was performed along with at least 10 studies, the results revealed that there was almost no publication bias in PLT (*p* = 0.895) (Figure 2A), but had certain publication bias in WBC (*p* = 0.095) (Figure 2B).

## Discussion

KDSS is a rare and serious complication of KD, which is even considered as a unique and serious KD subtype. KD is not difficult to diagnose, but the shock is not a common symptom in KD. And with few studies on KDSS, unclear mechanisms of its development as well as no predictive scoring standards, KDSS can be easily misdiagnosed. In this study, results revealed that the incidence of KDSS in children was slightly higher in boys (53%) than in girls (47%) (*p* = 0.48). Lin et al. (3) reported that the incidence of KDSS in children over 5 years of age was significantly higher than that of children under 5 (2.20 vs. 1.37%), and children aged ranging from 8 to 9 years showed the highest incidence of KDSS. Maddox et al. (4) also concluded that KDSS patients were more likely to occur in older children. In this study, the results showed that children with KDSS were older than children with KD, although some of the included studies might have been matched for age in order to be consistent with the baseline.

In the current studies, children with KDSS were more difficult to diagnose in early diagnosis. With no golden standard, diagnosis of KD was exclusionary that mainly depended on the clinical symptoms. Although children with KDSS had a longer duration of fever and a higher incidence of cervical lymphadenopathy than the children with KD, there was no significant difference in the comparison of the incidence of conjunctival injection, oropharyngeal changes, polymorphous rash, and extremity changes, fitting the results of IKD in this study (*P* = 0.20). KDSS mostly occurs in the acute phase of KD. Qiu et al. (22) reported that ~5 days (4–5.5 days) after the onset, KDSS was observed, which implied that shock might have occurred when the duration of fever was <5 days. The fever lasted for more than 5 days was a necessary criterion for the diagnosis of KD in the past. However, in the recent sixth revision of the KD diagnostic guidelines of the Japanese Circulation Society (23), the requirement for a specific fever range was removed that may probably reduce the misdiagnosis of KDSS.

The pathological changes in KD are systemic vascular inflammation, mainly occurring in small and medium-sized vessels. KDSS may result in damages to different systems due to an intensified systemic inflammatory storm. Children with KDSS had a significantly higher incidence of cardiovascular complications than KD, which mainly included coronary artery dilatation or coronary aneurysm, left ventricular dysfunction (decreased ejection fraction), acquired mitral regurgitation, and pericardial effusion. Gamez-Gonzalez et al. (24) reported that 44.6% (46/103) of children with KDSS had an ejection fraction <50% in the acute phase. Qiu et al. (22) also concluded that children with lower ejection fraction tended to have a higher risk, which could be used as a predictor for KDSS development. In addition, damage to other systems in children with KDSS had also been reported (24), which were 76.4% (47/63) in the digestive system, 31.7% (20/63) in the respiratory system, and 53.9% (34/63) in the nervous system.

Some laboratory examination indexes of children with KDSS had also significantly changed. The levels of WBC, NEUT%, and CRP in children with KDSS were significantly higher than non-shock KD patients, which suggested that KDSS children experienced a more serious systemic inflammatory response; higher level of ALT and AST might own to hepatocellular damage caused by inflammation; lower ALB and Na might be related with persistent capillary leakage caused by vasculitis, which might also promote the advancement of shock; lower PLT was a sign of diffuse intravascular coagulation, and also acted a risky factor for acute myocardial infarction and coronary artery aneurysm in KD (25).

The mechanism of IVIG resistance may be due to the fact that anti-cytokine antibodies are not sufficient to block the excess cytokines (26). Due to the more severe inflammatory response in KDSS children, IVIG resistance may occur, which often requires a second dose of IVIG or other additional treatments. Early use of glucocorticoids in children with KDSS may help to downregulate the inflammatory mediators and reduce capillary leakage (27). Anti-shock therapy should also be actively carried out at the same time to improve the state of systemic hypoperfusion and reduce organ damage. Vasoactive drugs, such as norepinephrine, epinephrine, dopamine, and dobutamine can be selected on an appropriate volume expansion. Besides, children with KDSS often have hypoalbuminemia. Albumin infusion can increase plasma colloid osmotic pressure and help maintain blood pressure stability. For children with lung, kidney, heart, and other organ failures, corresponding alternative treatments can be given, such as mechanical ventilation, plasma exchange, temporary pacemakers, and even extracorporeal membrane oxygenation (28–30). Through the above treatment, the condition of children with KDSS could be effectively controlled, and the shock can be quickly reversed to improve the prognosis.

Qiu et al. (22, 31) reported that indexes, such as age >3 years old, NEUT% >0.75, ALB <30 g/L, and NT-proBNP >11,000 pmol/L were independent risky factors for KDSS, and ejection fraction could be used as a predictor for KDSS. Schuster et al. (13) suggested that hyponatremia might be a risky factor for heart disease and shock. Shan et al. (32) identified troponin I, calcitoninogen, and NK cells as correlated factors for KDSS. Li et al. (16) reported a higher risk of KDSS in KD with IL-6 > 66.7 pg/ml, IL-10 > 20.85 pg/ml, and INF-γ > 8.35 pg/ml, and another study (33) showed that IL-6 and IL-10 levels were positively correlated with the occurrence time of KDSS shock, which was helpful for early diagnosis of KDSS.

However, the included studies were retrospective case-control studies, and some of the data reported in the literature were irregular or incomplete with potential publication bias, making the included sample size and data incomplete. The exploration of KDSS still needs prospective, large sample, and multicenter -joint researches.

## Conclusion

The symptoms of children with KDSS are basically in line with typical KD, yet with a longer duration of fever, more severe inflammatory indicators, and more cardiac abnormalities than non-shocked KD. These children may be resistant to the immunoglobulin treatments and need more anti-inflammatory and supportive treatments.

## Data Availability Statement

The original contributions presented in the study are included in the article/Supplementary Material, further inquiries can be directed to the corresponding author/s.

## Author Contributions

ZZ and YH conceived and designed the study. ZZ drafted the manuscript and YW edited the language. ZW and JT extracted and analyzed the data. XC, YL, ML, and CZ conducted the searching and screening of studies. YW and LW conducted the quality assessment. YM and LS made critical revisions. All authors contributed to the article and approved the submitted version.

## Conflict of Interest

The authors declare that the research was conducted in the absence of any commercial or financial relationships that could be construed as a potential conflict of interest.

## Publisher's Note

All claims expressed in this article are solely those of the authors and do not necessarily represent those of their affiliated organizations, or those of the publisher, the editors and the reviewers. Any product that may be evaluated in this article, or claim that may be made by its manufacturer, is not guaranteed or endorsed by the publisher.
