# Supplementary figures and images for: Clinical Features in Children With Kawasaki Disease Shock Syndrome: A Systematic Review and Meta-Analysis
Source: Front Cardiovasc Med. 2021 Sep 21;8:736352. doi: 10.3389/fcvm.2021.736352 (PMC8491834; doi:10.3389/fcvm.2021.736352)

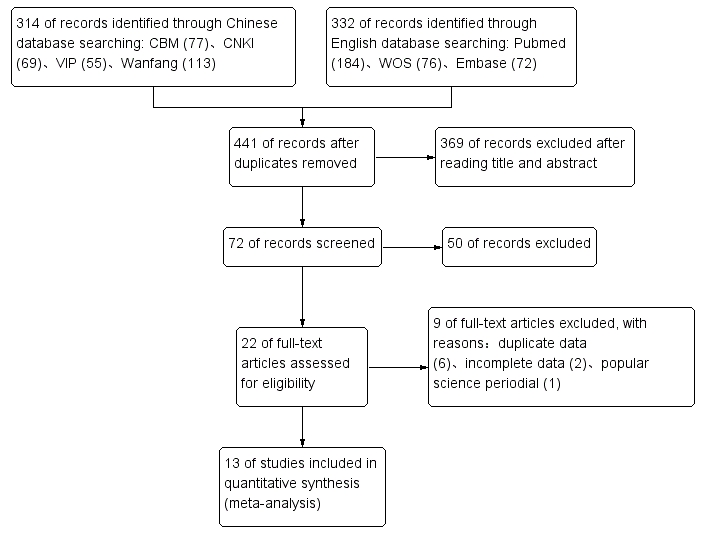

Supplement: Supplementary file 1 [file Data_Sheet_1.ZIP › Supplementary figures/Fig 1.jpg]

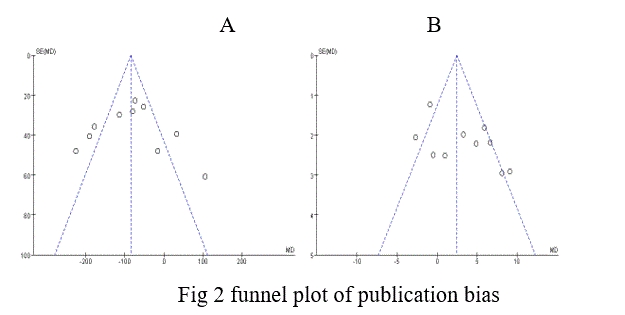

Supplement: Supplementary file 1 [file Data_Sheet_1.ZIP › Supplementary figures/Fig 2.jpg]

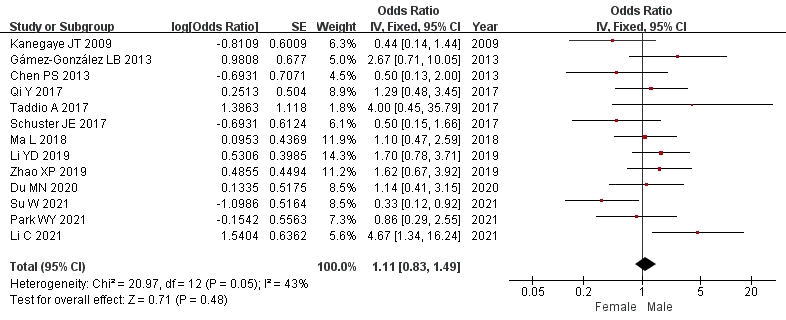

Supplement: Supplementary file 1 [file Data_Sheet_1.ZIP › Supplementary figures/Fig.S1.jpg]

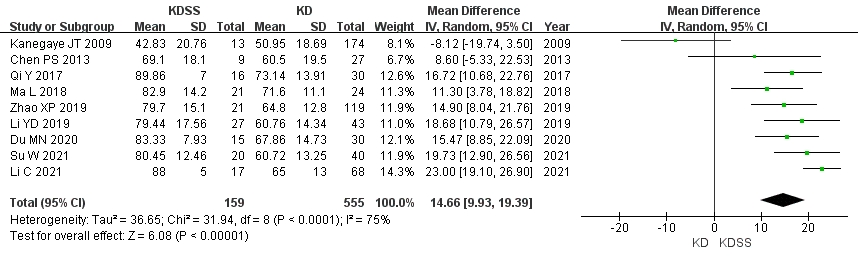

Supplement: Supplementary file 1 [file Data_Sheet_1.ZIP › Supplementary figures/Fig.S10.jpg]

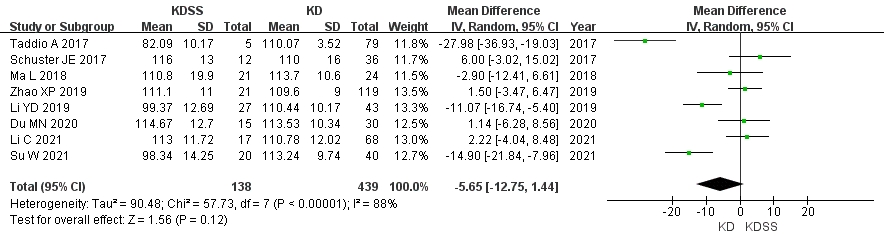

Supplement: Supplementary file 1 [file Data_Sheet_1.ZIP › Supplementary figures/Fig.S11.jpg]

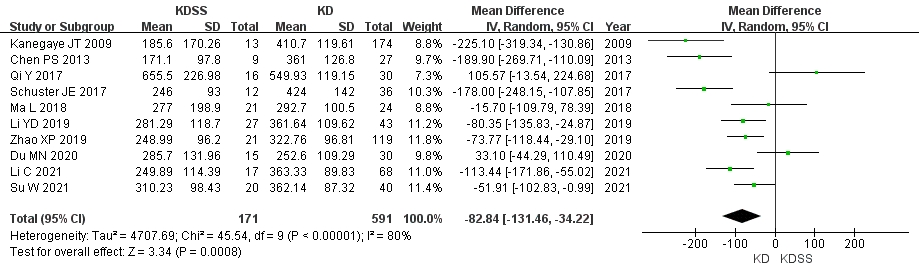

Supplement: Supplementary file 1 [file Data_Sheet_1.ZIP › Supplementary figures/Fig.S12.jpg]

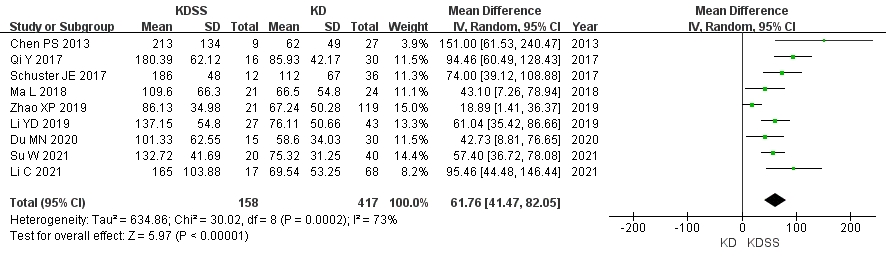

Supplement: Supplementary file 1 [file Data_Sheet_1.ZIP › Supplementary figures/Fig.S13.jpg]

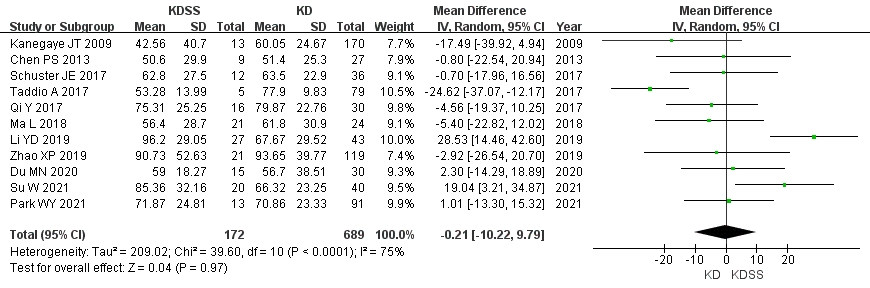

Supplement: Supplementary file 1 [file Data_Sheet_1.ZIP › Supplementary figures/Fig.S14.jpg]

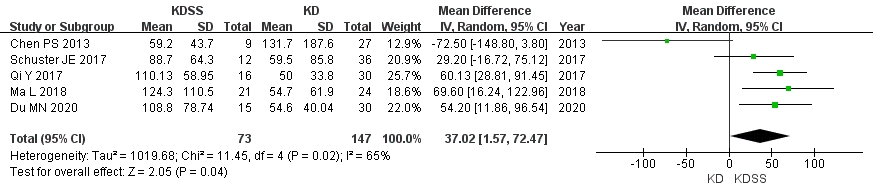

Supplement: Supplementary file 1 [file Data_Sheet_1.ZIP › Supplementary figures/Fig.S15.jpg]

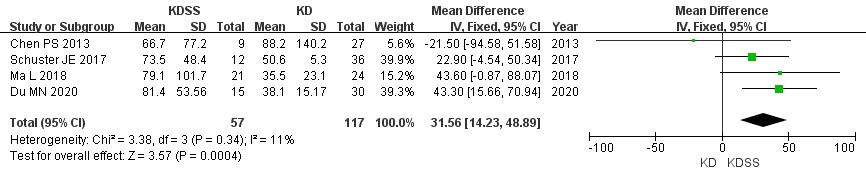

Supplement: Supplementary file 1 [file Data_Sheet_1.ZIP › Supplementary figures/Fig.S16.jpg]

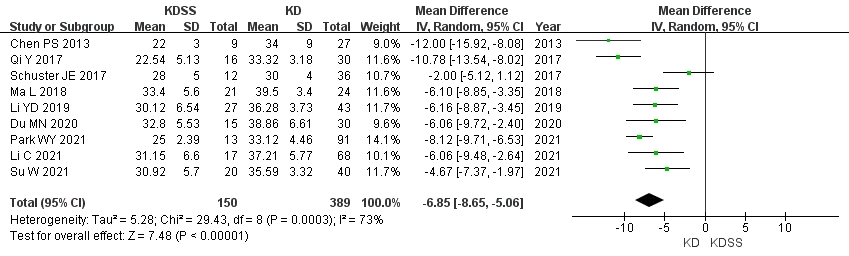

Supplement: Supplementary file 1 [file Data_Sheet_1.ZIP › Supplementary figures/Fig.S17.jpg]

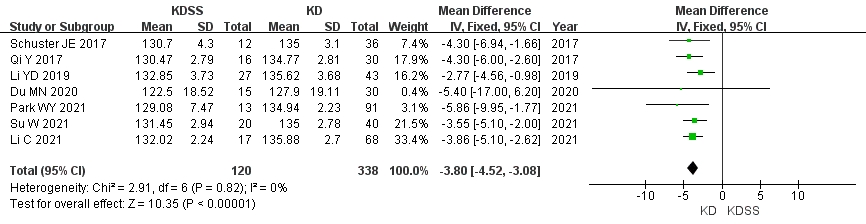

Supplement: Supplementary file 1 [file Data_Sheet_1.ZIP › Supplementary figures/Fig.S18.jpg]

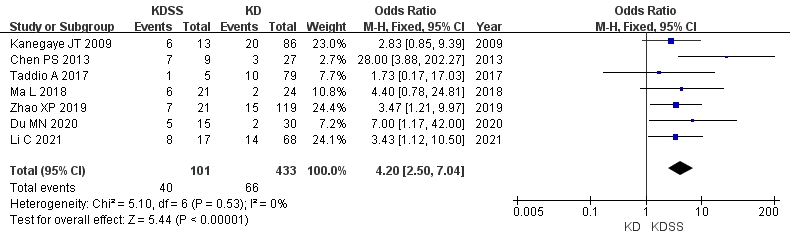

Supplement: Supplementary file 1 [file Data_Sheet_1.ZIP › Supplementary figures/Fig.S19.jpg]

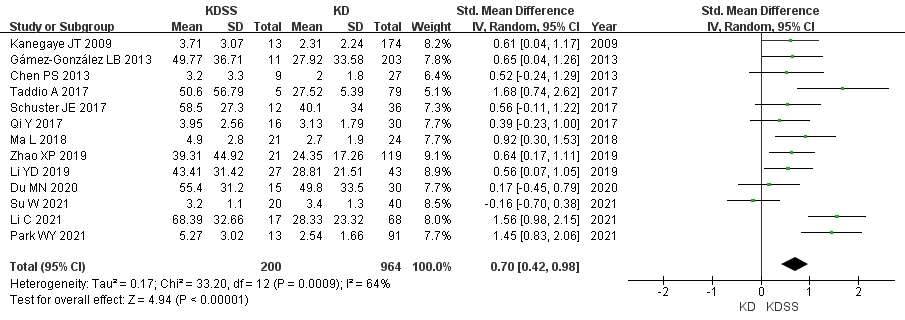

Supplement: Supplementary file 1 [file Data_Sheet_1.ZIP › Supplementary figures/Fig.S2.jpg]

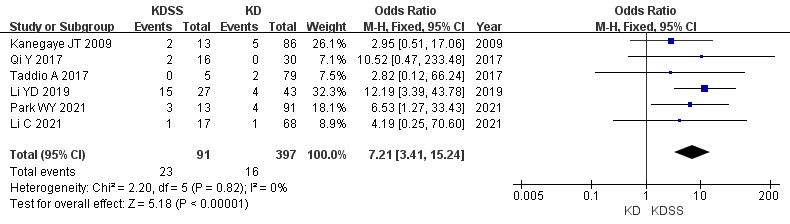

Supplement: Supplementary file 1 [file Data_Sheet_1.ZIP › Supplementary figures/Fig.S20.jpg]

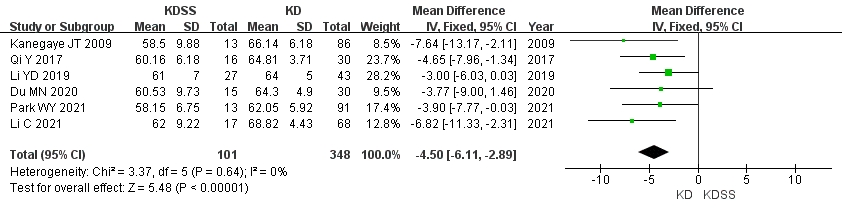

Supplement: Supplementary file 1 [file Data_Sheet_1.ZIP › Supplementary figures/Fig.S21.jpg]

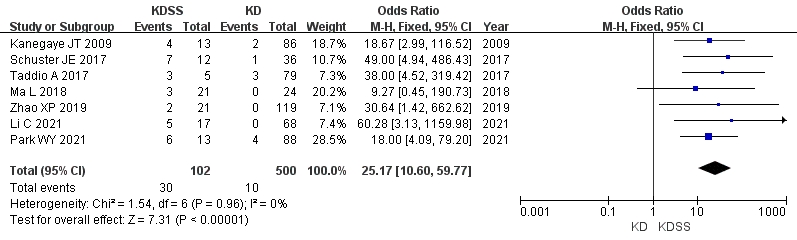

Supplement: Supplementary file 1 [file Data_Sheet_1.ZIP › Supplementary figures/Fig.S22.jpg]

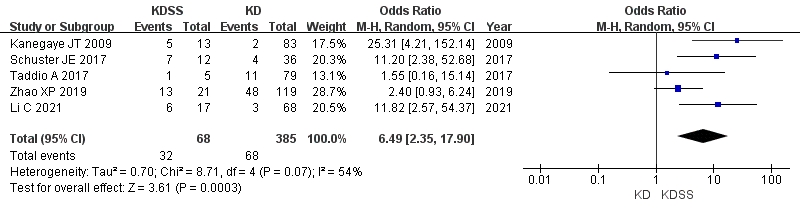

Supplement: Supplementary file 1 [file Data_Sheet_1.ZIP › Supplementary figures/Fig.S23.jpg]

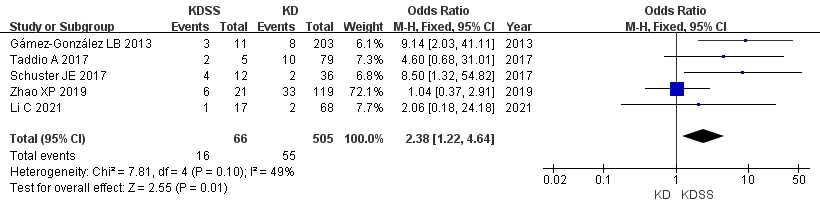

Supplement: Supplementary file 1 [file Data_Sheet_1.ZIP › Supplementary figures/Fig.S24.jpg]

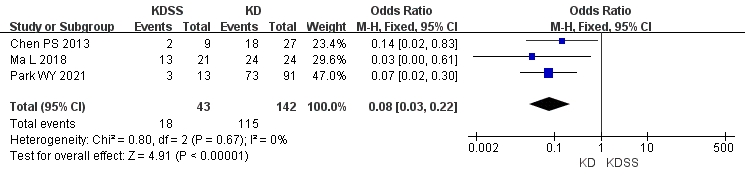

Supplement: Supplementary file 1 [file Data_Sheet_1.ZIP › Supplementary figures/Fig.S25.jpg]

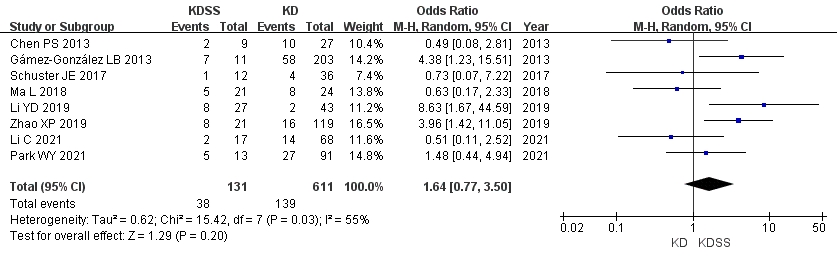

Supplement: Supplementary file 1 [file Data_Sheet_1.ZIP › Supplementary figures/Fig.S26.jpg]

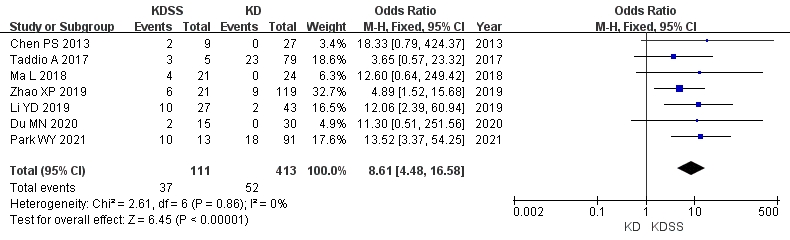

Supplement: Supplementary file 1 [file Data_Sheet_1.ZIP › Supplementary figures/Fig.S27.jpg]

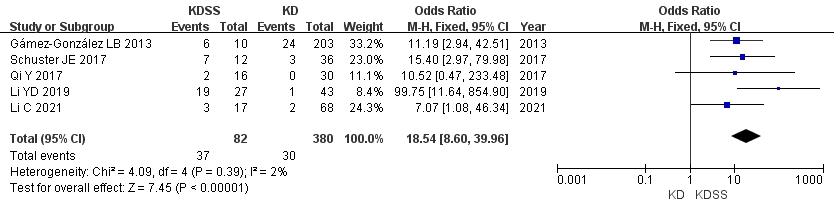

Supplement: Supplementary file 1 [file Data_Sheet_1.ZIP › Supplementary figures/Fig.S28.jpg]

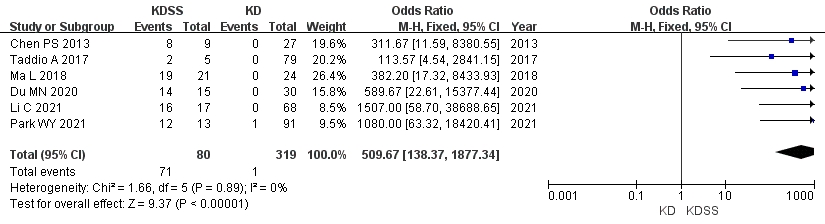

Supplement: Supplementary file 1 [file Data_Sheet_1.ZIP › Supplementary figures/Fig.S29.jpg]

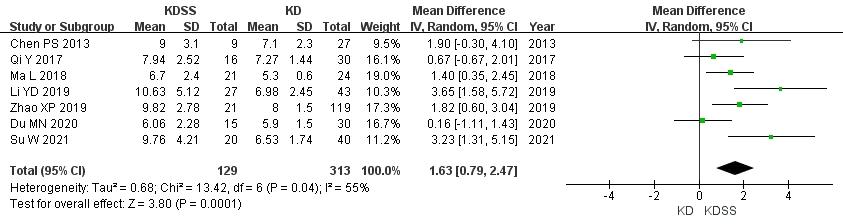

Supplement: Supplementary file 1 [file Data_Sheet_1.ZIP › Supplementary figures/Fig.S3.jpg]

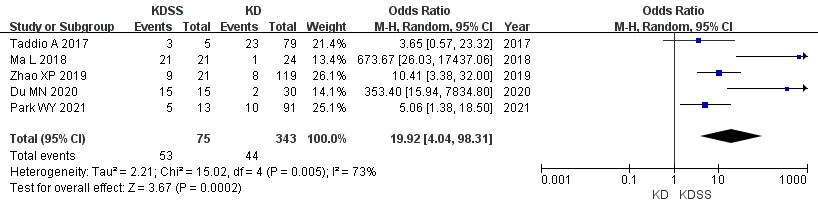

Supplement: Supplementary file 1 [file Data_Sheet_1.ZIP › Supplementary figures/Fig.S30.jpg]

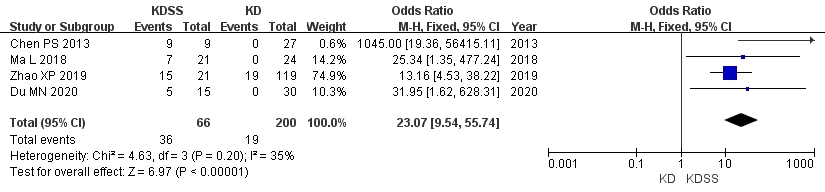

Supplement: Supplementary file 1 [file Data_Sheet_1.ZIP › Supplementary figures/Fig.S31.jpg]

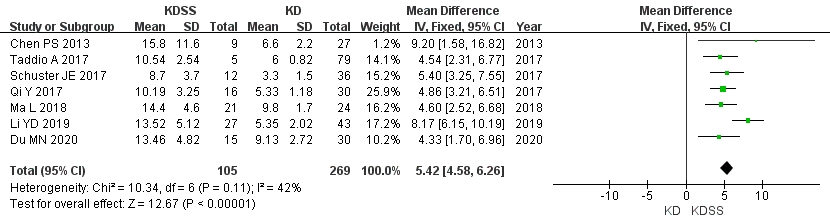

Supplement: Supplementary file 1 [file Data_Sheet_1.ZIP › Supplementary figures/Fig.S32.jpg]

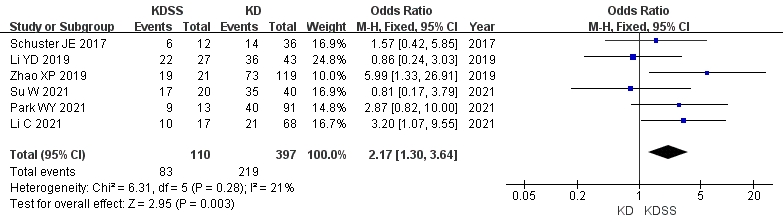

Supplement: Supplementary file 1 [file Data_Sheet_1.ZIP › Supplementary figures/Fig.S4.jpg]

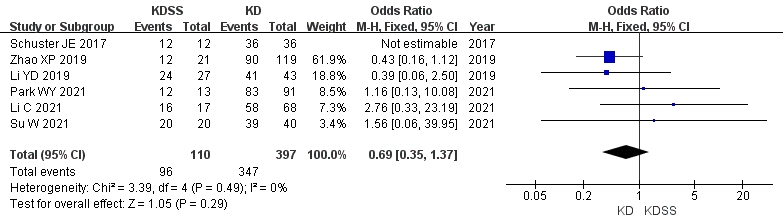

Supplement: Supplementary file 1 [file Data_Sheet_1.ZIP › Supplementary figures/Fig.S5.jpg]

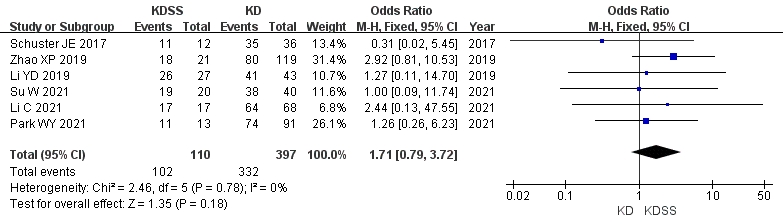

Supplement: Supplementary file 1 [file Data_Sheet_1.ZIP › Supplementary figures/Fig.S6.jpg]

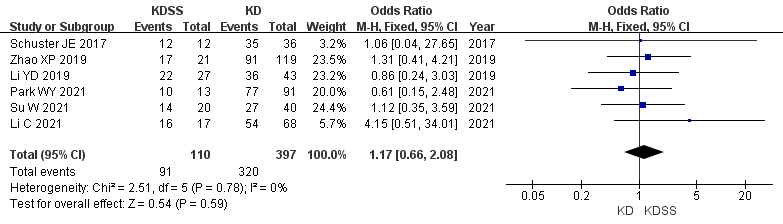

Supplement: Supplementary file 1 [file Data_Sheet_1.ZIP › Supplementary figures/Fig.S7.jpg]

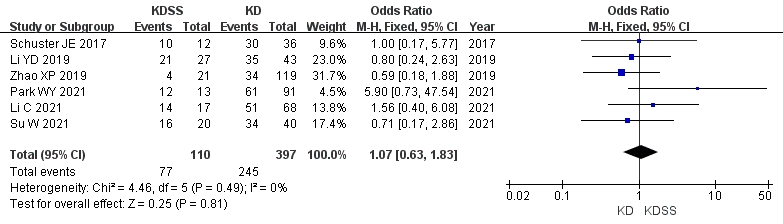

Supplement: Supplementary file 1 [file Data_Sheet_1.ZIP › Supplementary figures/Fig.S8.jpg]

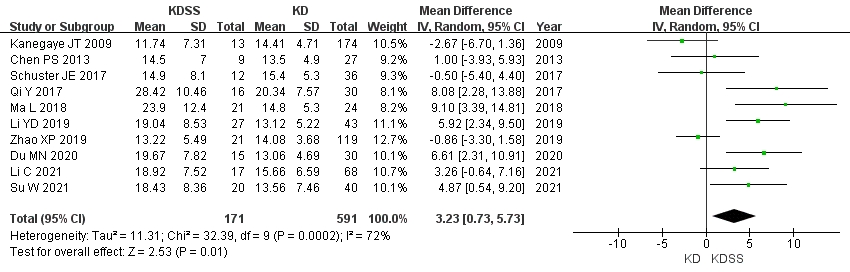

Supplement: Supplementary file 1 [file Data_Sheet_1.ZIP › Supplementary figures/Fig.S9.jpg]
